# Pulmonary Inflammatory Myofibroblastic Tumor in Children: A Case Report and Brief Review of Literature

**DOI:** 10.3389/fped.2018.00035

**Published:** 2018-02-27

**Authors:** Federica Camela, Marcella Gallucci, Emanuela di Palmo, Salvatore Cazzato, Mario Lima, Giampaolo Ricci, Andrea Pession

**Affiliations:** ^1^Department of Pediatrics, S. Orsola-Malpighi Hospital, University of Bologna, Bologna, Italy; ^2^Department of Mother and Child Health, Salesi Children’s Hospital, Ancona, Italy; ^3^Department of Pediatric Surgery, S. Orsola-Malpighi Hospital, University of Bologna, Bologna, Italy

**Keywords:** inflammatory myofibroblastic tumor, inflammatory pseudotumor, high-resolution computer tomography, histopathology assessment, anaplastic lymphoma kinase rearrangement

## Abstract

The inflammatory myofibroblastic tumor (IMT) is a rare lesion of unclear etiology and variable clinical course, consisting of a proliferation of fibroblasts and myofibroblasts, mixed with inflammatory cells. Synonyms of IMT are inflammatory pseudotumor and plasma cell granuloma reflecting the alleged inflammatory nature attributed to this lesion, even though this heterogeneity in the disease denomination is probably involved in a dispersion of the literature data. Among primary pulmonary neoplasms, it represents the most frequent endobronchial tumor of childhood and beyond the lung it has been described mainly in the bladder, mediastinum and mesentery. Despite having a tendency for local recurrence, the risk of distant metastasis is low. Clinical presentation depends on localization therefore lung peripheral lesions are often asymptomatic resulting in a delayed diagnosis. Radiological findings can suggest the diagnosis that must be confirmed by histopathology assessment. The tumor has been characterized by the application of immunohistochemical techniques, molecular biology and cytogenetics, which are very precious for the diagnosis. The therapeutic approach consists in the complete surgical excision of the lesion that normally ensures excellent survival. Due to the potential risk of recurrence, close clinical trial is indicated. To date only 24 cases of pulmonary IMT have been described, although the prevalence is probably higher. We present a case report of a 3-year-old girl with pulmonary IMT and a brief review of known literature cases in order to highlight the most common clinical presentations, the most useful diagnostic tools and therapeutic approach.

## Introduction

Primary pulmonary neoplasms in pediatric patients are uncommon diseases. Among these disorders, the inflammatory myofibroblastic tumor (IMT) represents 20% of all primary lung tumors and more of 50% of all benign masses (1, 2).

The World Health Organization (WHO) defines IMT as “a lesion composed of a proliferation of myofibroblastic spindle and stellate cells with abundant eosinophilic cytoplasm mixed with infiltrative plasma, inflammatory cells, lymphocytes and eosinophils” (3, 4).

The same entity was called in the past with other names; the most recognized ones were inflammatory pseudotumor (IPT), plasma cell granuloma, and postinflammatory tumor.

Inflammatory pseudotumor was considered as a reaction to an inflammatory insult (1, 5). It involve more often liver and biliary tract (31.8% of cases), head and neck (20.6%), lung (18.2%), abdomen (15.5%), and urogenitary system (7.4%) (6). Although it was considered in the past a non-neoplastic reactive inflammation, in 2006 WHO defined it an intermediary lesion with clinical recurrence and malignant potential (4).

These generic appellations reflect the alleged inflammatory and non-neoplastic nature attributed to these lesions, even though confusion regarding the distinction of these tumors from other entities such as the “inflammatory fibrosarcoma” (considered indistinguishable from the IMT), still remains (5, 7). This ambiguity in the denomination may be a factor favoring the dispersion of the literature data and then an underreporting.

## Case Report

We describe the case of a 3-year-old girl, born at 33 weeks of gestational age with a good neonatal adaptation. Starting from the age of 18 months, she presented with recurrent infections of lower and upper airways, which were often treated with antibiotic therapy. She came to our attention from another medical center.

In the previous 2 months, the child showed intermittent fever, loss of appetite and dry cough.

The chest X-ray (CXR) showed a rounded 2 cm diameter opacity in the left parahilar side, therefore she consecutively was treated with oral therapy with penicillin, cephalosporin of third generation and macrolide. Despite therapy, at first evaluation to our center the child was still having evening fever and poor general condition.

Chest auscultation revealed reduced air entry and fine crackles on the left hemithorax. The laboratory tests showed a stable increase of inflammatory markers: C-reactive protein 9.5 mg/dL (normal range <0.08 mg/dl), erythrocyte sedimentation rate 102 mm, leukocytes 11 × 10^6^/L (59% neutrophils, 32% lymphocytes, 8% monocytes, 0.4% eosinophils, and 0.7% basophils), hemoglobin 12 mg/dL, and platelet 809 × 10^3^/μL.

The nasal-pharyngeal samples culture and blood culture were both negative, including research for multidrug-resistant staphylococcus.

**Figure 1:**
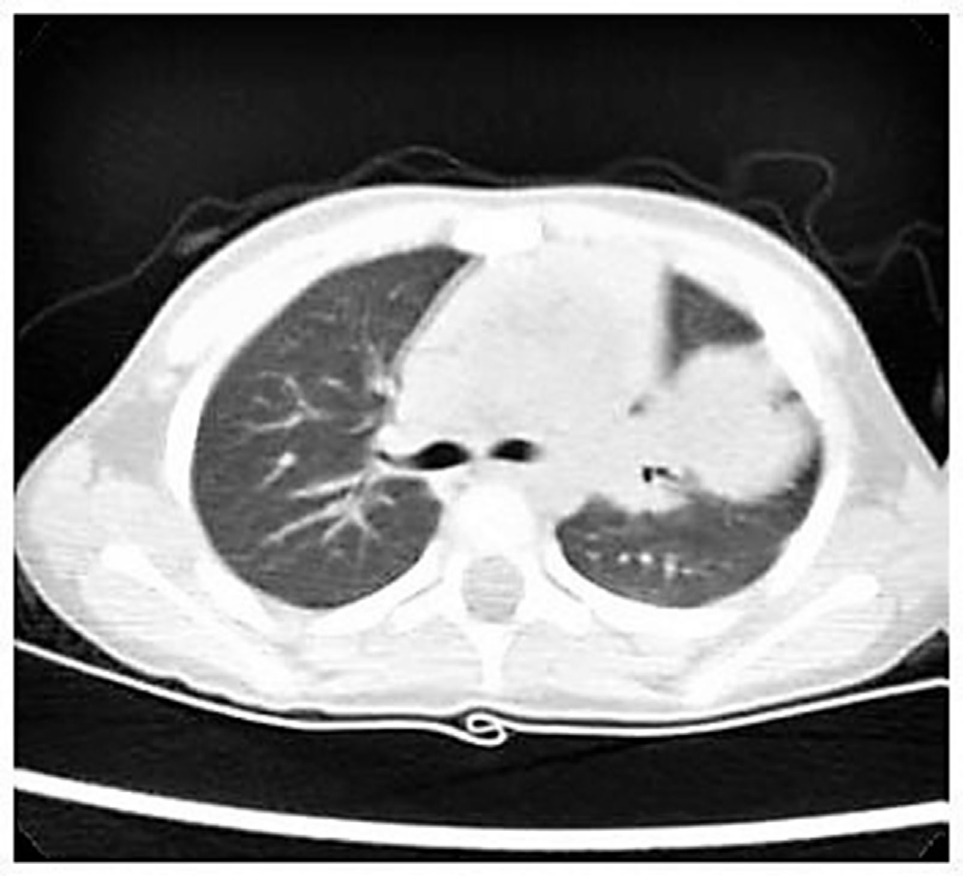
High-resolution computer tomography imaging showing a large lung lesion in the left parahilar side.

**Figure 2:**
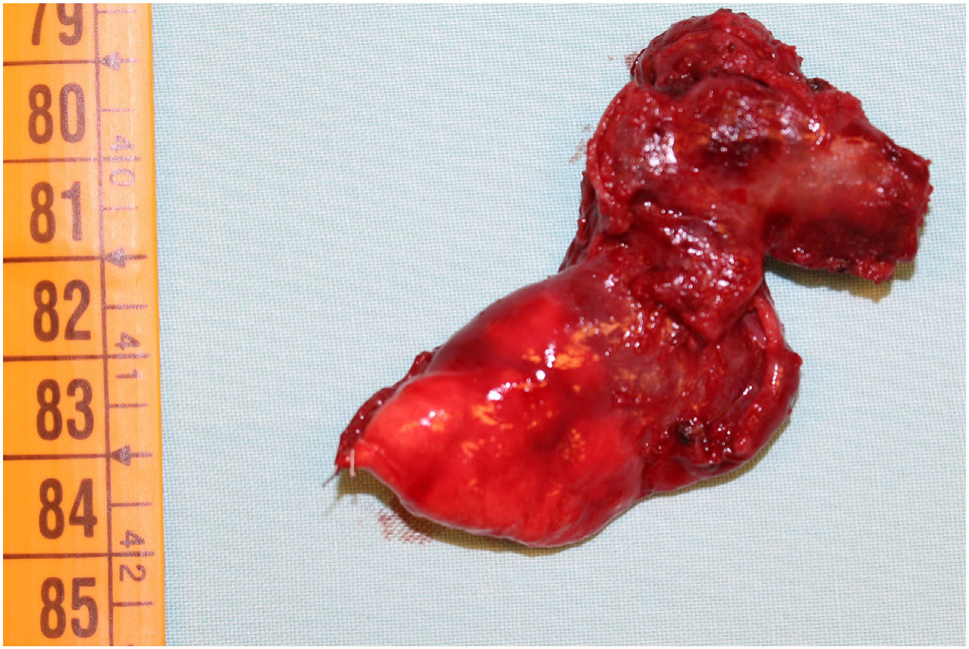
Surgical sample of lingula inflammatory myofibroblastic tumor in 3-year-old child.

Nevertheless, we started with empiric therapy with Piperacillin/Tazobactam and Linezolid. A few days later, because of persistent fever with altered laboratory and radiological findings, the child underwent a chest high-resolution computer tomography (HRCT) that showed a large lung lesion with thickened wall and fluid content at the lingula level (Figure 1). The mass diameter was about 35 mm. Suspecting a necrotizing pneumonia, surgical removal of the lesion was performed by atypical resection. The subsequent histological assessment showed an IMT positive for actin, anaplastic lymphoma kinase 1 (ALK1), and desmin (Figure 2). Subsequently, to achieve a complete removal of the lesion margins, the left upper lobectomy by thoracotomy was necessary.

The postoperative course was uncomplicated. After surgery, the clinical picture of the child gradually improved with remission of fever. At 6, 12, and 18 months of follow-up, she presented good clinical conditions and radiological evidence of atelectasis to the left lower lobe, compatible with recent surgery.

## Discussion

Although the spectrum of primary lung tumors in the childhood is the same as in adults, the lesions have different prevalence (8). IMT shows a high incidence in children and young people, representing 50% of primary benign lung tumor in infancy (1, 2).

Pulmonary IMT typically presents itself as a parenchymal nodule and occasionally as an endobronchial lesion (2). It has a tendency to grow slowly but can invade local structures (1).

The clinical picture depends primarily on the location of the tumor but most patients have a few respiratory symptoms so the diagnosis may be delayed.

Children with IMT may exhibit symptoms of chronic inflammation as a low-grade fever, weight loss, anemia, thrombocytosis, polyclonal hypergammaglobulinemia, and elevated sedimentation rate. Several cases are asymptomatic and are detected only incidentally on imaging studies.

Among patients with endobronchial lesions, symptoms of bronchial irritation such as cough and hemoptysis may be accompanied by chest pain (3).

In the current case, the previous clinical history, CXR and the presence of intermittent fever suggested at first an infectious disease. Nevertheless, the failure to respond to broad-spectrum antibiotics brought us to investigate the presence of a non-infectious lesion. The chest HRCT was decisive even though the diagnosis could not have been possible without histological assessment.

According to literature data, there are only 24 published cases of pediatric pulmonary IMT (the age thereof ranges from 3 to 13 years), even though the real incidence is presumed to be higher. Peripheral lung lesions appear to be more frequent than central and endobronchial tumors that may be present about in 10% of the cases resulting in bronchial obstruction and atelectasis (2).

Among the 24 described cases, the tumor grew in trachea in 5 patients (3 in carina; 20% of total) and was peripheral in 19 cases it (80% of cases). These peripheral masses were located in 11 cases (46%) in the left lobes and in 8 cases (33%) in the right lobes. At the onset, the mass was average on 4.5 cm of the diameter (1.4–12 cm); four subjects (17%) presented metastasis and secondary masses were discovered in one case after 11 months of follow-up. At the time of diagnosis, the IMT involved adjacent structures in five cases (21%): chest wall, main bronchus, mediastinum, hilum, or pulmonary artery (Table 1). In some of these cases, the patients came to clinicians’ attention with symptoms related to the local effects of the mass such as stridor or a history of wheezing (5).

**Table 1:** Review of 25 known cases of pediatric pulmonary IMT, including our case: diagnostic tools, histological assessment, and immune-histochemical analysis of lung bioptic samples.

	Patients	Diagnostic tools	Histological assessment	Immuno-histochemical analysis
El-Desoky et al. (9)	F, 9 y	HRCT: mass in left main bronchus	Compatible with IMT^a^	n.d.
Johnson et al. (10)	M, 7 y	CXR: solitary right lung mass	Compact spindle cells mixed with abundant plasma cells and lymphocytes. Extensive collagenous deposition with metaplastic bone and numerous calcific deposits.Metastatic IMT involving the entire visceral and parietal pleural surfaces	Positive for vimentin
Hammas et al. (11)	M, 3 y	HRCT: left lower lobe mass	Proliferation of regular spindle cells arrayed in fascicles, mixed with lymphocytes, plasma cells and eosinophils	Positive for ALK1, SMA, and H-caldesmon
Prahbu et al. (12)	F, 4.5 y	CXR and HRCT: large circumscribed mass in the left hemithorax abutting the left anterolateral chest wall; calcification and areas of necrosis within	Not available data	n.d.
	F, 6 y	CXR and HRCT: large left upper lobe mass with areas of necrosis and calcification within	Spindle cell proliferation with focally hyalinized, collagenized stroma and dense chronic inflammation with several plasma cells	Positive for vimentin and SMA
	F, 8 y	CXR and HRCT: mass in left hemithorax abutting the left pulmonary artery; similar left paravertebral lesion	Compatible with IMT^a^	Not available data
Brodlie et al. (13)	F, 9 y	CXR: well-circumscribed solitary right apical mass	Spindle cells with an inflammatory infiltrate	Not available data
	F, 15 y	HRCT: well-circumscribed left main bronchus mass	Compatible with IMT^a^	Positive per ALK
	F, 11 y	Bronchoscopy	Compatible with IMT^a^	Not available data
Sacco et al. (14)	F, 6 y	HRCT: round paratracheal mass in the right middle portion of the mediastinum	Fusiform, fibroblast-like cells and spindle cells surrounded by a collagenous stroma, growing in interlacing fascicles with prominent storiform pattern	Positivity for vimentin, SMA, and clusterin
Ochs et al. (15)	F, 5 y	HRCT: mass in left main bronchus with bronchial obstruction; atelectasis of the left haemitorax	Intrabronchial IPT (fibrohistiocytoma type): benign spindle cells with submucosal tissue with a palisading pattern (arrow) and admixed leukocytes	Not available data
Fernández del Castillo Ascanio et al. (16)	F, 5 y	HRCT: left lung mass extending to anterolateral mediastinum and abutting the left pulmonary artery and left main bronchus; 1 contralateral lung lesion and 7 brain masses	Compatible with IMT^a^	Not available data
Lindemans et al. (17)	M, 6 m	HRCT: solid left upper lobe mass	Highly proliferative myofibroblastic spindle cells whit an infiltrate of mononuclear cells, macrophage foam cells and sporadic necrotic debris	Not available data
	F, 6 y	Ultrasound: solid right lower lobe mass; central calcification	Solid area of proliferating fibroblast with collagen and fibrosis	Not available data
Lizarbe et al. (18)	M, 8 y and 10 m	HRCT: tracheal mass abutting the left main bronchus; left lung collapsed	Mixoid areas with abundant vascularization, fibroblast cells, epithelioid cells, and histiocytes mixed with inflammatory cells	Positive per vimentina, desmina, SMA, CK AE1-AE3, ALK, protein S-100, and HHV8
Breen et al. (19)	F, 11 y	MR: endobronchial mass located in the left main stem bronchus	Compatible with IMT^a^	Not available data
Takayama et al. (20)	M, 4 y	HRCT and MR: well-circumscribed right upper lobe mass	Homogeneously whitish mass with spindled myofibroblasts, chronic inflammatory infiltrate, and lymphoid aggregates	Positive for SMA
	F, 7 y	HRCT and MR: well-circumscribed round mass in the left lower lobe	Homogeneously red tissue, composed of spindled myofibroblasts with chronic inflammatory infiltrate and lymphoid aggregates	Positive for SMA
Venizelos et al. (21)	M, 13 y	HRCT: mass at bifurcation of the trachea	Spindle shaped cells with ovoid or round-shaped nuclei, sparse chromatin and eosinophilic cytoplasm. Nuclear pleomorphism, minimal and rare mitotic figures	Positive for vimentin, ALK and CD68. A few cells exhibited reactivity for SMA and muscle specific actin
Sivanandan et al. (22)	F, 9 y	Bronchoscopy	Squamous lining epithelium and a spindle cell lesion in the sub-epithelial region	Positivity for SMA, vimentin
Hoseok et al. (23)	M, 4 y	Bronchoscopy	Proliferation of spindle-shaped fibroblasts and myofibroblasts arrayed in fascicles with some storiform architecture.	Positive for SMA and vimentin
Pichler et al. (24)	F, 12 y	CXR and HRCT: large and solid homogeneous lesion with contrast enhancement	Solid and well encapsulate, different level of fibrosis and inflammation of normal plasma cells as well as some macrophages, lymphocytes, and eosinophils	Not available data
Chan et al. (25)	F, 7 y	HRCT: large mass enveloping the right main bronchus	Fibrous tissue with areas of necrosis, extensive lymphocytic infiltration, and plasma cells	Not available data
Prasad et al. (26)	M, 12 y	CXR: round homogenous left-paracardiac mass	Compatible with IMT^a^	Not available data
Current case, 2017	F, 3 y	HRCT: large lung lesion with thickened wall and fluid content at the lingula level	Spindle cell proliferation with inflammatory infiltrate	Positive for actin, ALK1, and desmin

*y, years; m, months; w, weeks; d, days; h, hours; n.d., not done, immuno-histochemical analysis not performed; CXR, chest X-ray; HRCT, high-resolution computer tomography; MR, magnetic resonance; ALK, anaplastic lymphoma kinase; SMA, alfa-smooth muscle actin; CK, cytokeratins (AE 1–3)*.

*^a^Histological features not specified by the authors*.

Inflammatory myofibroblastic tumor can be sometimes detected as incidental finding on a routine CXR (27). In all cases, at presentation patients had fever, respiratory distress, arthralgia, clubbing, night sweat, vomiting, and hemoptysis. At the onset, fever and cough were the commonest symptoms (Table 2).

**Table 2:** Frequency of symptoms in pediatric pulmonary IMT, including our case.

Symptoms at presentation	Symptoms rate^a^
Cough	12 (48%)
Fever	11 (44%)
Respiratory distress	10 (40)
Anemia	9 (36%)
Weight loss	5 (20%)
Stridor	3 (12%)
Chest pain	2 (8%)
Recurrent respiratory infections	2 (8%)
Arthralgia	2 (8%)
Clubbing	2 (8%)
Sweat	2 (8%)
Wheezing	2 (8%)
Exertional dyspnea	1 (4%)
Seizure	1 (4%)
Hemoptysis	1 (4%)
Vomiting	1 (4%)
Asymptomatic	1 (4%)

*The table lists the cases of literature except one in which the author did not specify symptoms related to right-side pneumonia (20). One asymptomatic case is reported in a child with incidental diagnosis made by CXR (10)*.

*^a^Number of patients; the parentheses indicate the percentage of symptoms*.

Data collected from the literature show that central IMT are recognized earlier, probably due to the resulting symptoms (acute obstruction of the trachea, carena, or main bronchus). On the contrary, peripheral parenchymal tumors cause more misleading and nonspecific symptoms leading to delayed diagnosis.

Including our patient, in nine cases the children presented with variable degree of anemia. They also showed laboratory findings of chronic inflammation such as leukocytosis, hypergammaglobulinemia, elevated platelet count, increase of erythrocyte sedimentation rate, and C-reactive protein (12, 14, 15, 17, 18, 24).

The anatomopathological differential diagnosis is wide and must be conducted on the clinical picture, morphological alterations, and the immune phenotype of spindle cells.

Differential diagnosis must include foreign body aspiration, infectious disease (Aspergillum and Mycobacterium) but also necrotizing pneumonia and spindle cell neoplasms (1).

The histological assessment is essential to rule out pseudo-lymphomas, malignant lymphomas, lymphoid hyperplasia and sarcomas such as leiomyosarcoma, fibrosarcoma, rhabdomyosarcoma, myxoid sarcoma, malignant fibrous histiocytoma, and gastrointestinal stromal tumor (12, 14, 15, 17, 18, 24, 27, 28).

Chest X-ray usually reveal a peripheral lesion or nodule of variable size (diameter usually ranges from 1.2 to 15 cm,) more often localized in the lower lobes (29). Rarely, patients may have multiple lesions (30).

Among the literature cases, 16/24 subjects underwent HRCT often showing solid masses with homogeneous or heterogeneous enhancement (Table 1). These lesions appear as single, well-defined, lobulated mass and frequently contain punctate calcifications that mimics malignant tumors (20).

Positron emission tomography/computed tomography is highly sensitive but has low specificity for IMT. It could be useful to evaluate the response to treatment in patients not eligible for surgery (31).

Bronchoscopy is usually performed when there exists a suspect of endoluminal lesion. It is a useful diagnostic and therapeutic tool since it has both a function of removing tumoral obstruction and providing biopsy samples (13, 19).

Moreover, due to local recurrence risk, bronchoscopy may have a potential role in the follow-up even though future evidences are necessary to define the timing of endoscopic revaluation.

Among all pediatric known cases, only three subjects underwent magnetic resonance imaging (MRI): in two cases the chest MRI confirmed the HRCT findings and MRI was performed only in one case in place of HRCT (19, 20).

Takayama et al. suggest that radiological presentation and contrast-enhancement in pediatric IMT are variable and nonspecific. Moreover, calcifications within the tumor are present more often in children than in adults (these were reported in seven children; 29% of cases) (20).

In association with lesions, there could be pneumothorax, pleural effusion, atelectasis, and rarely cavitations and lymphadenopathies (Table 1).

Molecular rearrangements on chromosome 2p23 were detected in several cases of IMTs (in about 34–56% of both pulmonary and extrapulmonary IMTs). This locus is the site of human *ALK* gene, which codes for a tyrosine kinase receptor called ALK. *ALK* rearrangements are identified by immunohistochemical analysis (fluorescence *in situ* hybridization) using monoclonal ALK-1 antibody and seems to be highly specific for these lesions, even though it does not seem to be a sensitive marker in children (32, 33). Furthermore, ALK-positivity does not seems to be related to recurrence (1, 5, 33, 34).

Abnormal karyotypes of tumor cells, like aneuploidy, were found in 16% of cases (32, 33). *RET* gene rearrangement, *EML4-ALK* inversion, and fusions of other kinases gene have also been implicated (35).

Alaggio et al. in a recent report found that cytologic atypia and positive ALK status are more frequent in aggressive tumors, whereas metastatic tumors are negative for ALK.

Among previous pediatric cases described, positivity to vimentin (29%), alpha-smooth muscle actin (alpha-SMA) (21.6%), ALK (16.6%), and other markers like desmin, cytokeratins (AE 1–3), CD34, protein S-100, and HHV8 was reported (32–34).

Treatment is primarily a complete but conservative surgical excision (3). This approach is necessary to prevent recurrence (1, 28). An appropriate histologic assessment should be obtained before the surgery (needle biopsy by bronchoscopy), in order to avoid an unnecessarily procedure (28).

Among all cases, 15 patients underwent open lung surgery, 2 resections by laser, 1 surgery by thoracoscopy, and 1 child was treated with bronchoscopic excision. In one case treated with open surgery, coexistent cerebral masses were detected and they required chemotherapy (16).

The most appropriate surgical approach (such as use of the wedge resection, lobectomy, and pneumonectomy) depends on the specific dimension and tumor localization, its relationship with the surrounding structures and the surgical equipment experience (36).

Partial resection may be necessary in cases where it is not possible to remove the lesion because of invasion of the vital structures. Among these cases, chemotherapy may be an alternative option for patients who have microscopic or macroscopic residual disease, although the results are controversial (32).

Described therapy for expanded masses were vinblastine plus methotrexate (14).

Although systemic steroids showed some beneficial effects in IMT, Sacco et al. found that the use of Prednisone may favorite the progression of lesion probably due to immunosuppression, therefore the authors suggest caution in the use of this drug in IMT (15).

In two cases without metastasis or local invasion, chemotherapy (methotrexate or cyclophosphamide associated with prednisone) was started as primary treatment (13).

In others two cases, the clinicians decided to undertake a therapy with only corticosteroids without evidence of recurrence at follow-up (15). Another case was treated only with COX-2 inhibitor after evidence of unresectable mass, with complete resolution at 8 months of follow-up (25).

Among all patients, only three children had tumor recurrence (10, 12, 17).

Although recurrence is described as a rare phenomenon (14% of pulmonary IMT) a close follow-up should be made for the early recognition of tumor relapse. In addition, the local invasion at diagnosis was highly correlated with local relapse (9, 11, 37).

In our case, we decided to perform a complete surgical excision of the lesion followed by left upper lobectomy; nevertheless, to date we do not know the rate of survival after surgery versus others managements. The most recent evidence in young adult (mean age 33 years) suggests a 5- and 10-year disease-free survival of 89% after complete resection (23, 26, 37).

## Take Home Message

- Pulmonary IMT is an uncommon disease but with significant morbidity among the pediatric population.
- Children may show non-specific and variable symptoms therefore they often underwent several antibiotic treatments before diagnosis.
- Radiological techniques are useful in suspected lesions but the diagnosis must be confirmed by histopathological assessment.
- Surgical complete resection is the treatment of choice.
- Because of the potential malignant behavior of these tumors, a close follow-up should be done to recognize the recurrences earlier.
- Due to the rarity of the disease among the pediatric population, it may be useful for the future to include all known cases in a national registry in order to improve knowledge of the disease and encode the best diagnostic and therapeutic approach.

## Ethics Statement

The authors state that “written informed consent was obtained from the parents of the patient for the publication of this case report.”

## Author Contributions

GR coordinated the writing group. FC and MG performed the literature review. All authors critically reviewed the manuscript, read, and approved the final version.

## Conflict of Interest Statement

The authors declare that the research was conducted in the absence of any commercial or financial relationships that could be construed as a potential conflict of interest.

## Abbreviations

IMT, inflammatory myofibroblastic tumor; IPT, inflammatory pseudotumor; HRCT, high-resolution computer tomography; ALK1, anaplastic lymphoma kinase 1; MRI, magnetic resonance imaging; CXR, chest X-ray.
